# Optical measurement of gating pore currents in hypokalemic periodic paralysis model cells

**DOI:** 10.1242/dmm.049704

**Published:** 2023-06-27

**Authors:** Tomoya Kubota, Satoe Takahashi, Risa Yamamoto, Ruka Sato, Aya Miyanooto, Reina Yamamoto, Kosuke Yamauchi, Kazuaki Homma, Masanori P. Takahashi

**Affiliations:** ^1^ Department of Clinical Laboratory and Biomedical Sciences, Division of Health Sciences, Osaka University Graduate School of Medicine, Suita, Osaka, Japan; ^2^ Department of Otolaryngology - Head and Neck Surgery, Feinberg School of Medicine, Northwestern University, Chicago, IL 60611, USA; ^3^ The Hugh Knowles Center for Clinical and Basic Science in Hearing and Its Disorders, Northwestern University, Evanston, IL 60208, USA

**Keywords:** Hypokalemic periodic paralysis, Voltage-gated sodium channel, Gating pore current, Proton

## Abstract

Hypokalemic periodic paralysis (HypoPP) is a rare genetic disease associated with mutations in *CACNA1S* or *SCN4A* encoding the voltage-gated Ca^2+^ channel Cav1.1 or the voltage-gated Na^+^ channel Nav1.4, respectively. Most HypoPP-associated missense changes occur at the arginine residues within the voltage-sensing domain (VSD) of these channels. It is established that such mutations destroy the hydrophobic seal that separates external fluid and the internal cytosolic crevices, resulting in the generation of aberrant leak currents called gating pore currents. Presently, the gating pore currents are thought to underlie HypoPP. Here, based on HEK293T cells and by using the Sleeping Beauty transposon system, we generated HypoPP-model cell lines that co-express the mouse inward-rectifier K^+^ channel (mKir2.1) and HypoPP2-associated Nav1.4 channel. Whole-cell patch-clamp measurements confirmed that mKir2.1 successfully hyperpolarizes the membrane potential to levels comparable to those of myofibers, and that some Nav1.4 variants induce notable proton-based gating pore currents. Importantly, we succeeded in fluorometrically measuring the gating pore currents in these variants by using a ratiometric pH indicator. Our optical method provides a potential *in vitro* platform for high-throughput drug screening, not only for HypoPP but also for other channelopathies caused by VSD mutations.

## Teaser

**Summary:** Hypokalemic periodic paralysis model cells that allow the fluorometric evaluation of gating pore currents in a multi-well-plate format.

## INTRODUCTION

Hypokalemic periodic paralysis (HypoPP) is a rare skeletal muscle disease characterized by intermittent episodes of muscle weakness and paralysis of various severity, which is caused by a dysfunction of voltage-gated ion channels regulating the excitability of the sarcomere (Cannon, 2015). The two causative genes identified to date are *CACNA1S*, which encodes the voltage-gated Ca^2+^ channel Cav1.1 associated with HypoPP type1 (HypoPP1) and *SCN4A*, which codes the voltage-gated Na^+^ channel Nav1.4 associated with HypoPP type 2 (HypoPP2) (Statland et al., 2018). Both diseases are inherited in an autosomal dominant fashion and their clinical manifestations are indistinguishable. For years, it was unclear how mutations in these two distinct genes yield an identical disease phenotype.

Most HypoPP-associated missense changes are found at the positively charged arginine residues of segment 4 (S4) in Cav1.1 and Nav1.4, which play an essential role in voltage sensing in these voltage-gated ion channels (Bezanilla, 2008). Recent structural and functional studies point to a pathological mechanism in which mutations of arginine residues within the S4 voltage-sensing domain (VSD) destroy the hydrophobic seal (also known as hydrophobic plug, gating-charge transfer center, or hydrophobic gasket) between the external fluid and the internal cytosolic crevices, thus generating an aberrant ionic leak, i.e. the gating pore current (Campos et al., 2007; Lacroix et al., 2014; Li et al., 2014; Pan et al., 2018; Tao et al., 2010). Ion species passing through the gating pore are determined by both the properties and the position of a VSD mutation. In the Shaker Kv channel, replacement of the outer-most S4 arginine (R) residue – also called ‘first gating charge’ (R1) – with histidine (H) within the S4 has been reported to generate a proton-specific gating pore through the Grotthuss hopping mechanism (Starace and Bezanilla, 2004). A similar mechanism accounts for the proton-specific permeation caused by substitution of the first gating charge-bearing arginine to histidine in domain II of Nav1.4 (Struyk and Cannon, 2007), whereas mutations of the other arginine residues result in gating pores that are also permeable for other monovalent cations, such as Na^+^ and K^+^ (Sokolov et al., 2007, 2010; Struyk et al., 2008). Currently, gating pore currents due to noncanonical passage of ions through Cav1.1 or Nav1.4 channels are presumed to underlie HypoPP (Cannon, 2010; Matthews et al., 2009; Sokolov et al., 2007; Struyk and Cannon, 2007). Consistently, of twenty-two HypoPP-associated *CACNA1S* and *SCN4A* variants identified to date, fourteen have experimentally been demonstrated to exhibit gating pore currents (Tables S1 and S2).

At present, acetazolamide (Matthews et al., 2011) and dichlorphenamide (Jitpimolmard et al., 2020; Sansone et al., 2016, 2021; Statland et al., 2018) are approved by the United States Food and Drug Administration (FDA) for treating HypoPP. Both are known to inhibit carbonic anhydrase, but it remains to be elucidated how this inhibition affects HypoPP. Uncertainty also exists regarding why acetazolamide worsens HypoPP in some patients (Torres et al., 1981). Treatment with bumetanide, an inhibitor of the Na^+^/K^+^/Cl^−^ co-transporters (Wu et al., 2011, 2013a,b), has also been promising as it facilitates recovery from the paralytic state in a HypoPP mouse model. In addition, small molecules that directly target the cause of HypoPP – i.e. the gating pore – are of great interest. For the Nav1.4 variant, 1-(2,4-xylyl) guanidium has been shown by Sokolov and colleagues to block the gating pore currents in a *Xenopus* oocyte expression system; however, as its IC_50_ is within the millimolar range it is unsuitable for clinical use. (Sokolov et al., 2010). Search for additional candidate drugs that specifically block the gating pore currents of HypoPP-associated Cav1.1 or Nav1.4 variants with high affinities has been hindered by the lack of suitable experimental systems, as neither the *in vitro* cut-open voltage-clamp (COVC) techniques in *Xenopus* oocytes (Groome et al., 2014; Sokolov et al., 2007, 2010; Stefani and Bezanilla, 1998; Struyk et al., 2008; Struyk and Cannon, 2007) nor the *in vivo* assessment of drug efficacy in HypoPP mouse models (Wu et al., 2011, 2012) are ideal for high-throughput screening. The *Xenopus* oocyte expression system has been robustly used for pharmacological characterization of gating pore currents (Männikkö et al., 2018), and whole-cell recording in oocytes can be automated by, for example, using the Roboocyte system (Leisgen et al., 2007). However, since maturation and membrane targeting of some disease-associated variants are known to be temperature sensitive (Yoon et al., 2008), it is worth pursuing the use of a mammalian cell expression system for producing mutated mammalian proteins of interest under the physiological condition.

**Fig. 1.**
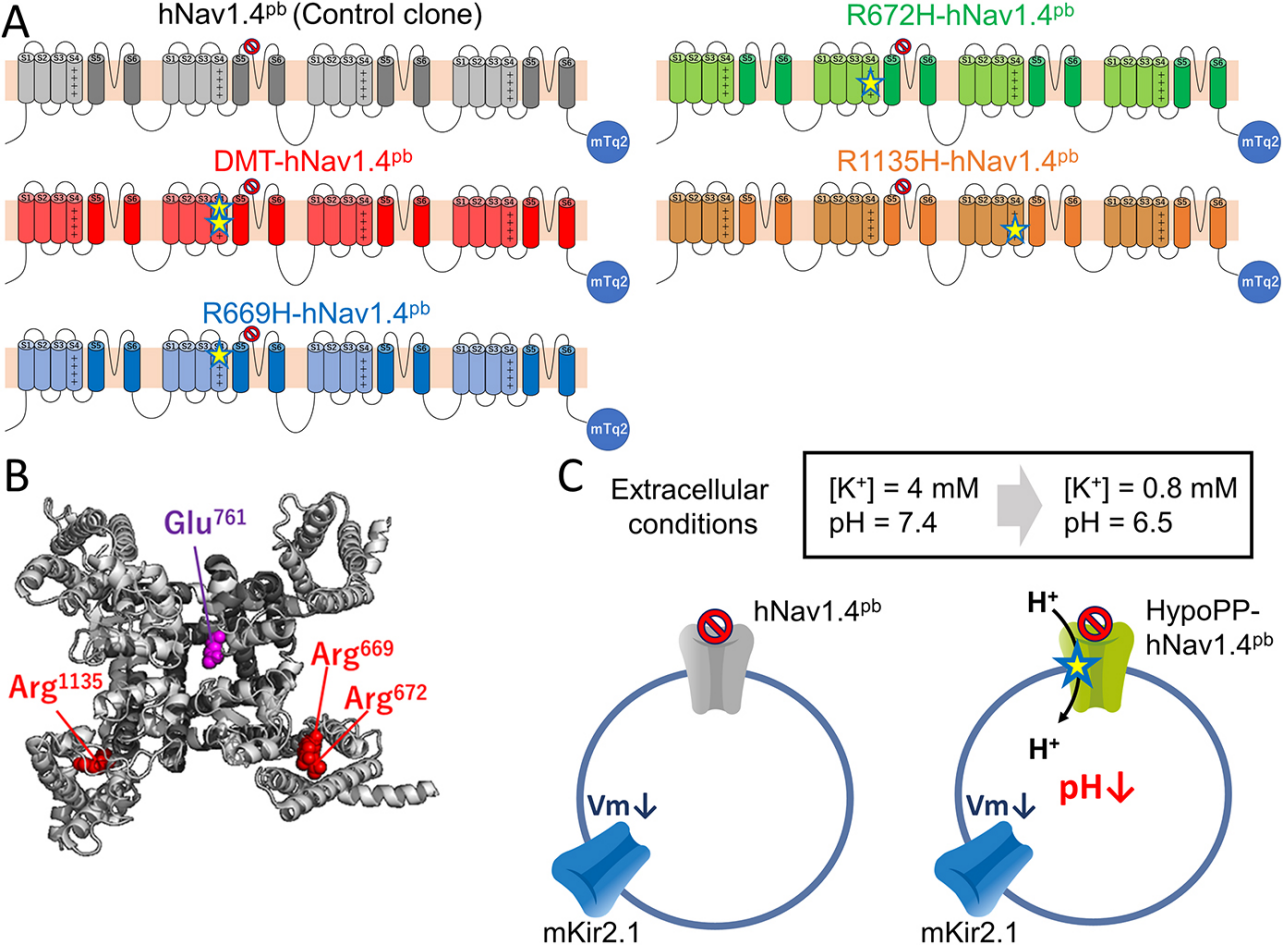
**Experimental design.** (A) Schematic representation of five human Nav1.4 constructs used in this study, i.e. hNav1.4^pb^ (pore-blocked control; grey), and the four HypoPP-hNav1.4^pb^ constructs DMT-hNav1.4^pb^ (R669H/R672H double mutation; red), R669H-hNav1.4^pb^ (R669H single mutation; blue), R672H-hNav1.4^pb^ (R672H single mutation; green), R1135H-hNav1.4^pb^ (R1135H single mutation; orange). The canonical ionic pore-blocking (pb) mutation E761K was introduced to all hNav1.4 constructs (hNav1.4^pb^) to facilitate detection of small gating pore currents and is indicated by the red ‘No Waiting’ sign. mTurquoise2 (mTq2) was attached to the C-termini in all constructs to facilitate the visual analysis of protein expression. The location of the HypoPP-associated mutations R669H, R672H and R1135H is indicated by yellow stars. DMT indicates the R669H/R672H double mutation. (B) Structure of human Nav1.4 (PDB: 6AGF) (Pan et al., 2018). The location of amino acid residues mutated in this study are indicated in purple (Glu^761^) or red (Arg^669^, Arg^672^, Arg^1135^). (C) Schematic of HypoPP model cells established in this study and the experimental procedure used for optical measurement of proton-gating pore currents. Co-expressed were either hNav1.4^pb^ control (gray), one of its four mutant constructs (green; yellow star indicates a HypoPP-associated mutation), the Na^+^ channel β1 subunit (not shown) and mouse Kir2.1 (blue). Before the optical proton transport assay, levels of extracellular K^+^ and pH values were reduced (from 4 mM to 0.8 mM and pH 7.4 to pH 6.5, respectively) to augment the electrochemical gradient that drives H^+^-mediated gating pore currents. Changes in intracellular proton (H^+^) levels, resulting from gating pore currents, were fluorometrically monitored using the ratiometric pH indicator SNARF-4F.

In this study, we sought to establish a plate-reader-based fluorometric assay to non-electrophysiologically evaluate gating pore currents in mammalian cell lines that heterologously express HypoPP2-associated human Nav1.4 (hNav1.4) channel variants (Fig. 1A,B). The mouse inward-rectifier K^+^ channel (mKir2.1) was co-expressed in these cell lines to hyperpolarize the membrane potential of the cells to drive gating pore currents in a pathophysiologically relevant extracellular environment (Fig. 1C). We show here that gating pore currents are fluorometrically detectable in some HypoPP model cells, thereby providing proof-of-concept for this *in vitro* optical system to be used for high-throughput drug screening.

## RESULTS

### Generation of HEK293T-based stable cell lines

We focused on the three HypoPP2-associated Nav1.4 missense variants p.R669H, p.R672H and p.R1135H (hereafter referred to as R669H, R672H and R1135H, respectively), to detect proton-gating pore currents (Bulman et al., 1999; Jurkat-Rott et al., 2000; Matthews et al., 2009). To facilitate detection of small gating pore currents, we introduced the E761K mutation (see Fig. 1B for the location of Glu^761^) – which corresponds to the canonical channel pore-blocking E942K mutation in rat Nav1.2 (Schlief et al., 1996) – to human Nav1.4 (hereafter referred to as hNav1.4^pb^). The cyan fluorescent protein mTurquoise2 (mTq2) was attached to the hNav1.4^pb^ C-terminus to visualize expression (Fig. 1A). By using the P2A self-cleaving peptide, we also added the Na^+^ channel β1 subunit after mTq2 to form the hNav1.4 channel complex (see Materials and Methods). cDNAs coding for all these molecular components were then cloned into the pSBtet-Pur expression vector (Kowarz et al., 2015). This design allowed expression of mTq2-tagged hNav1.4 and the β1 subunit at similar levels in a doxycycline-inducible manner. Based on hNav1.4^pb^, we then generated four additional hNav1.4 constructs harboring the single mutations R669H (R669H-hNav1.4^pb^), R672H (R672H-hNav1.4^pb^) or R1135H (R1135H-hNav1.4^pb^), or the double mutation R669H/R672H (DMT-hNav1.4^pb^). Although the double mutant has not been found in patients, it was included in our study as it is presumed to exhibit a larger gating pore current according to a previous study (Capes et al., 2012). In other words, we used DMT-hNav1.4^pb^ as a positive control for detecting the gating pore current. pSBtet-Pur vectors expressing the above hNav1.4^pb^ constructs and the β1 subunit were transfected into HEK293T cells to establish doxycycline-inducible cell lines. Our pSBtet-Pur vector plasmids were also transfected into HEK293T cells that constitutively express mouse Kir2.1 (mKir2.1) cloned in the pSBbi-Bla vector (addgene #60526), to establish doubly selected cell lines used in our optical assay (Fig. 1C). The K^+^ conductance mediated by Kir2.1 greatly hyperpolarizes the membrane potential (Vm) of the cell and is essential for driving inward gating pore currents (see below). Moreover, Kir2.1 is of physiological relevance in HypoPP, as both Nav1.4 and Kir2.1 are expressed in skeletal muscles.

### Electrophysiological characterization of HypoPP2 model cell lines

In general, mammalian cell line-based expression systems are preferable over oocytes when characterizing mammalian proteins, as they are more amenable to high-throughput screening formats. However, owing to the larger cell surface that allows an increased expression of membrane proteins, oocyte systems have preferentially been used for studying small currents, such as gating pore currents. As such, only few studies have so far reported gating pore currents in cell lines, including that of Nav1.5 in HEK293T cell (Moreau et al., 2015a,b), or in induced pluripotent stem cell-derived cardiomyocytes (Moreau et al., 2018). Thus, it is crucial to establish experimental conditions under which small gating pore currents can be readily detected in cell lines that express HypoPP-associated Nav1.4 channels.

**Fig. 2.**
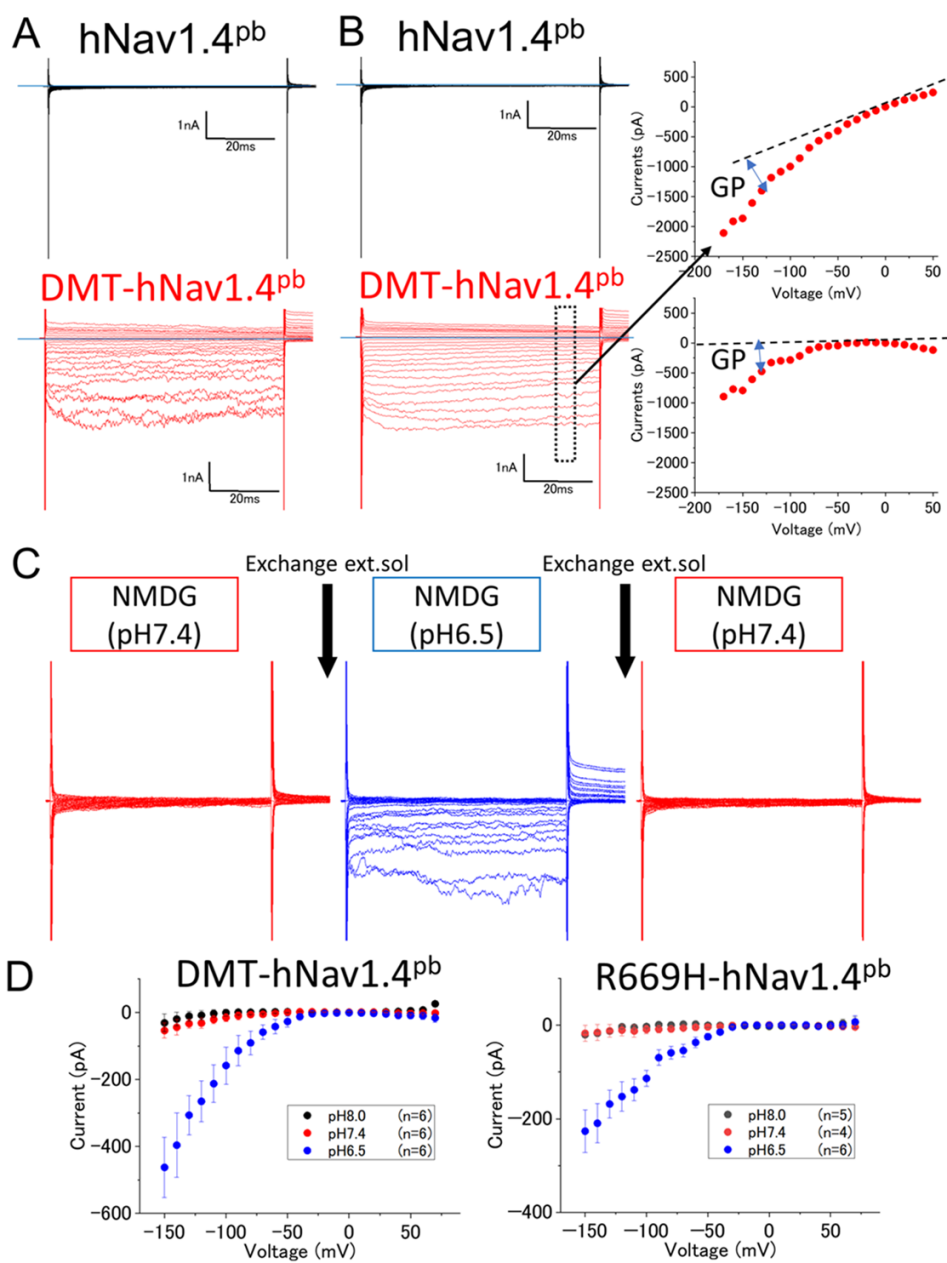
**Gating pore current measurements.** (A,B) Representative raw data of voltage-dependent whole-cell currents collected from HEK293T cells expressing hNav1.4^pb^ (top) or DMT-hNav1.4^pb^ (bottom) in MES-buffered Ringer's solution (A) or N-methyl-D-glucamine (NMDG)-based solution (B). External and internal pH values were 6.5 and 7.4, respectively. Horizontal blue lines indicate a current level of zero. Pulse protocol details to measure the current–voltage relationship between −150 mV and +70 mV. Blue horizontal lines indicate 0 mV; ΔV = −10 mV. Scale bars indicate time in ms (*x*-axis) and electric current in nA (*y*-axis). The boxed area in panel B indicates the range (65−68.5 ms) of the averaged data used to generate the current-over-voltage (I-V) curve, i.e. the current–voltage relationship, pointed to by the black arrow (top right panel in B). The graph below (bottom right) shows the true (net) gating pore current after correcting the data shown in the curve above by using off-line subtraction. Data for off-line subtraction were obtained between −20 mV and +20 mV. Blue double-headed arrows indicate the magnitude of the net gating pore (GP) current. (C) Transmembrane currents from one cell expressing DMT-hNav1.4^pb^, obtained by using whole-cell voltage clamp technique. pH values of NMDG-based extracellular solution (ext.sol) were changed by perfusion from pH 7.4 to pH 6.5 and then reverted to pH 7.4. (D) Current–voltage relationships of DMT-hNav1.4^pb^ (left) and R669H-hNav1.4^pb^ (right) determined in NMDG-based extracellular solutions at pH 6.5 (blue), pH 7.4 (red), and pH 8.0 (black). Fig. S1 shows the same data sets but corrected for cell membrane capacitance (current density).

We first examined whether gating pore currents are electrophysiologically detectable in cells expressing DMT-hNav1.4^pb^. To this end, whole-cell currents were measured in MES-buffered Ringer's solution at pH 6.5 (hereafter simply referred to as Ringer's solution) by applying the whole-cell voltage-clamp configuration (Fig. 2A). The transmembrane currents were minimal in cells expressing hNav1.4^pb^ across voltage due to the E761K pore-blocking mutation and the absence of gating pore conductance expected for this negative control. By contrast, as expected, cells expressing DMT-hNav1.4^pb^ exhibited large inward gating pore currents below −70 mV. To identity the ion species responsible for the observed gating pore currents, we repeated the experiment by using a solution in which Na^+^ and K^+^ were replaced with N-methyl-D-glucamine (NMDG). As shown in Fig. 2B, similar transmembrane currents were measured when using NMDG solution, suggesting that influx of H^+^ accounts for the observed inward gating pore currents. Note that representative raw data of currents (Fig. 2A and B) contain intrinsic linear leak currents. After the measurements, data of electrical currents were corrected by subtracting their linear background for the leak that was determined from current data between −20 mV and +20 mV (Fig. 2B, right panels). We also examined the dependence of inward currents induced at hyperpolarized membrane potentials on transmembrane proton gradients by using recordings from the same cell before (pH 7.4) and after (pH 6.5) extracellular perfusion. As shown in Fig. 2C, hyperpolarization-induced currents measured in a cell expressing DMT-hNav1.4^pb^ increased upon lowering the pH from 7.4 to 6.5 in a reversible manner, suggesting that the observed inward currents are proton-based gating pore currents mediated by the mutated Nav1.4 protein. We also found that inward currents did not increase upon raising the external pH from 7.4 to pH 8.0 in cells expressing DMT-hNav1.4^pb^ or R669H-hNav1.4^pb^ (Fig. 2D; Fig. S1), confirming proton-based gating pore currents.

**Fig. 3.**
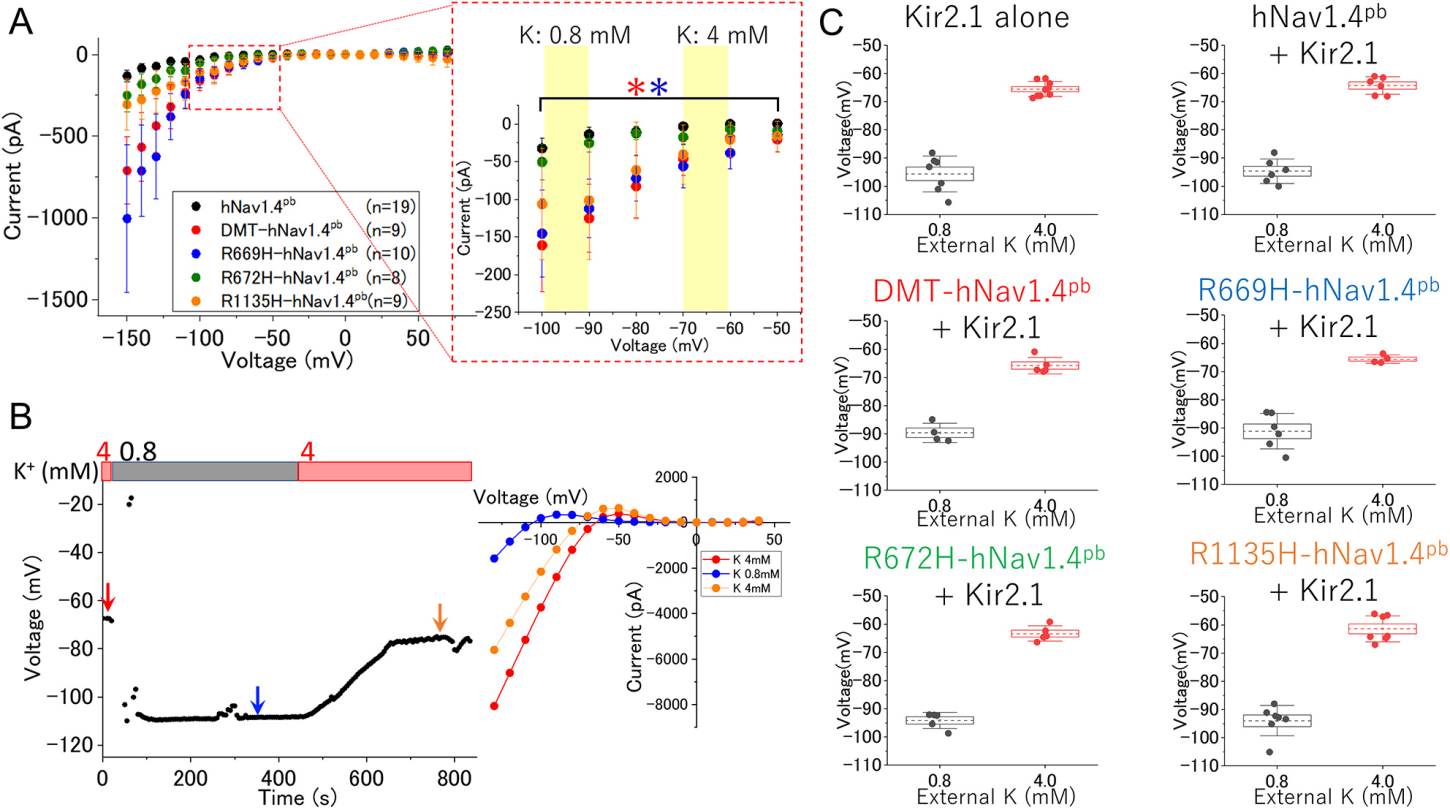
**Comparison of gating pore currents and analysis of the effects expression of Kir2.1 has on the membrane potential in HypoPP model cells.** (A) Voltage-dependent whole-cell currents of cells expressing hNav1.4 constructs kept in Ringer's solution. Off-line leak subtractions were conducted manually as exemplified in Fig. 2B, right panels. The mean±s.e.m. is shown for each hNav1.4^pb^ construct as indicated. *n*, number of replicates. The boxed area is shown enlarged on the right, showing data between −50 mV and −100 mV. Yellow rectangles indicate cell membrane potentials attained by changes in the extracellular K^+^ concentration (from 4 mM to 0.8 mM). Differences in current–voltage relationships of hNav1.4 constructs compared to that of hNa1.4^pb^ control and assessed by linear regressions for data between −50 mV and −100 mV are shown in Fig. S2. Statistical significances were found for DMT-hNav1.4^pb^ (red; *P=0.0003) and R669H-hNav1.4^pb^ (blue; *P=0.0022). The mean±s.e.m. is shown as indicated. (B) Time-dependent measurement of the membrane potentials of an mKir2.1-expressing HEK293T cell by whole-cell current clamping. Extracellular K^+^ concentration was changed by perfusion from 4 mM to 0.8 mM and back to 4 mM (indicated by red and gray bars). Colored arrows indicate three different time points at which the current–voltage relationships were determined by voltage-clamp recordings (red: 4 mM K^+^; blue: 0.8 mM K^+^; orange: 4 mM K^+^). The reversal potentials determined from these current–voltage profiles are shown in the right panel and agree with the membrane potentials directly determined using the Vm track mode (left panel). (C) Summaries of resting membrane potentials of HEK293T cells expressing mKir2.1 alone (top left) or together with hNav1.4^pb^ constructs as indicated, determined in external solution containing either 0.8 mM K^+^ (black circles) or 4.0 mM K^+^ (red circles). Dashed lines indicate mean values with boxes around them indicating the ±s.e.m.; error bars indicate the ±s.d.

The presence of gating pore currents was further examined in Ringer's solution at pH 6.5 and compared between HypoPP2-associated missense variants, i.e. R669H-hNav1.4^pb^, R672H-hNav1.4^pb^ and R1135H-hNav1.4^pb^, together with the pore-blocked control hNav1.4^pb^ (Fig. 3A). We found that all variants exhibited aberrant leak currents at negative potentials, although to various degrees. Specifically, R672H-hNav1.4^pb^ as well as R1135H-hNav1.4^pb^ showed relatively small gating pore currents and their magnitudes were statistically indistinguishable from those of hNav1.4^pb^, whereas R669H-hNav1.4^pb^ exhibited DMT-hNav1.4^pb^-like large gating pore currents (Fig. 3A; Fig. S2). These results show that – at least for some HypoPP2-associated Nav1.4 variants – gating pore currents are inducible and detectable in a HEK293T-based heterologous expression system when the membrane potential (Vm) is sufficiently low (below −70 mV).

### Optical proton transport assay

Having found that gating pore currents are mediated by H^+^ and electrophysiologically detectable – at least for R669H – and DMT-hNav1.4^pb^ in HEK293T cells (Figs 2 and 3), we decided to monitor these gating pore currents by using not electrophysiology techniques but, instead, a pH-sensitive fluorophore, since intracellular pH values should change upon in- or efflux of H^+^. Fluorometric assays, such as the one used by us, are readily compatible with a plate reader-based recording format; however, the resting membrane potential of HEK293T cells is typically around −20 mV, which is unlikely to drive gating pore currents for any of the hNav1.4 variants (Figs 2 and 3A). Since the inward-rectifier K^+^ channel (Kir2.1) plays a main role in hyperpolarizing the resting membrane potential of the skeletal muscle fibers (DiFranco et al., 2015), we generated a stable cell line constitutively expressing mouse Kir2.1 (mKir2.1) and examined its electrophysiological properties using whole-cell patch clamp recording (Fig. 3B). Current clamp recording, i.e. voltage tracking, confirmed a greatly hyperpolarized resting membrane potential in mKir2.1-expressing cells and its dependence on the extracellular K^+^ concentration (Fig. 3B). Specifically, the resting membrane potentials of cells expressing mKir2.1 were determined to be −65.5±2.7 or −95.6±6.4 mV (mean±s.d., *n*=9) in solutions containing 4 or 0.8 mM K^+^, respectively. Similar results were obtained in cells stably co-expressing hNav1.4^pb^ and mKir2.1 (−64.3±3.1 mV or −94.7±4.3 mV), DMT-hNav1.4^pb^ and mKir2.1 (−65.8±2.9 mV or −89.6±3.4 mV), R669H-hNav1.4^pb^ and mKir2.1 (−65.6±1.4 mV or −91.1±6.3 mV), R672H-hNav1.4^pb^ and mKir2.1 (−63.2±3.1 mV or −93.9±3.2 mV), and R1135H-hNav1.4^pb^ and mKir2.1 (−61.4±4.6 mV or −94.0±5.4 mV) with 4 mM or 0.8 mM K^+^, respectively (Fig. 3C). These results indicate that the physiologically relevant low resting membrane potential can be attained in HEK293T cells by co-expressing mKir2.1 but also be readily manipulated by changing the external K^+^ concentration (Fig. 3B, C) that is crucial for non-electrophysiologically driving inward gating pore currents in a plate reader-based optical recording format.

**Fig. 4.**
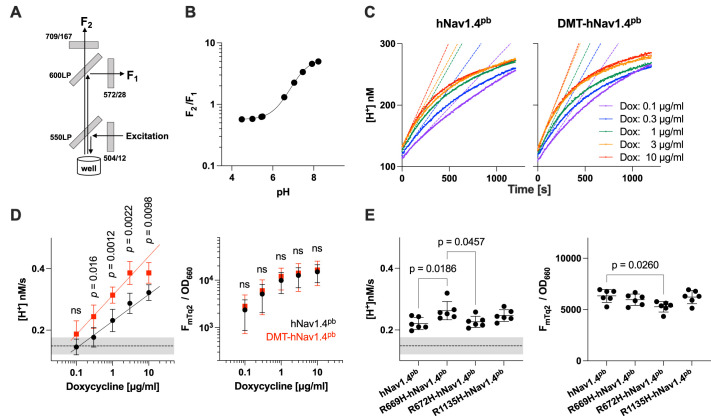
**Proton transport assay in HypoPP-model cells.** (A) Schematic of the optical configuration used for the assay. Gray boxes indicate filters and dichroic mirrors. (B) Response of the ratiometric fluorescence indicator SNARF-4F to different pH values. F_1_ and F_2_ are the fluorescence intensities of SNARF-4F measured using the fluorescence band-pass filters as shown in A. The ratio between F_1_ and F_2_ (F_2_/F_1_) is plotted against the pH values. (C) Ratiometric pH data obtained in H^+^ transport assays of cells expressing hNav1.4^pb^ or DMT-hNav1.4^pb^ induced by concentrations of doxycycline as indicated. Dashed lines indicate initial rates, solid lines indicate single exponential curve fits. (D,E) Summary of H^+^ transport rates (left panels) and protein expression (right panels) for hNav1.4^pb^ and DMT-hNav1.4^pb^ (D), and for R669H-hNav1.4^pb^, R672H-hNav1.4^pb^ or R1135H-hNav1.4^pb^ induced by 1 µg/ml doxycycline (E). Horizontal dashed lines indicate the basal transport activity of cells only expressing mKir2.1 (negative control), gray underlays indicate the ±s.d. Expression of hNav1.4^pb^ constructs was determined by measuring mTq2 fluorescence (F_mTq2_) corrected for the optical density of cells at 660 nm (OD_660_). Student's *t*-test. ns, not significant (*P*>0.05). One-way ANOVA with Tukey's multiple comparison test. For *P*≥0.05, comparisons were not shown. Error bars indicate the mean±s.d.

For our fluorometric proton transport assay, the ratiometric pH indicator SNARF-4F was loaded into cell lines stably co-expressing the mTq2-tagged hNav1.4^pb^ constructs, the β1 subunit and mKir2.1. The pH-sensitive fluorescence of SNARF-4F was simultaneously measured at F_1_ and F_2_ using the 96-well plates in a high-speed plate reader (Fig. 4A, B). This simultaneous fluorometry made our assay immune to any change of fluorescence intensities caused by photobleaching and/or cell movements between measurements. The use of cell suspensions (∼10^5^ cells/well) also enabled greatly increased signal-to-noise ratios to sensitively detect subtle pH changes caused by small gating pore currents.

**Fig. 5.**
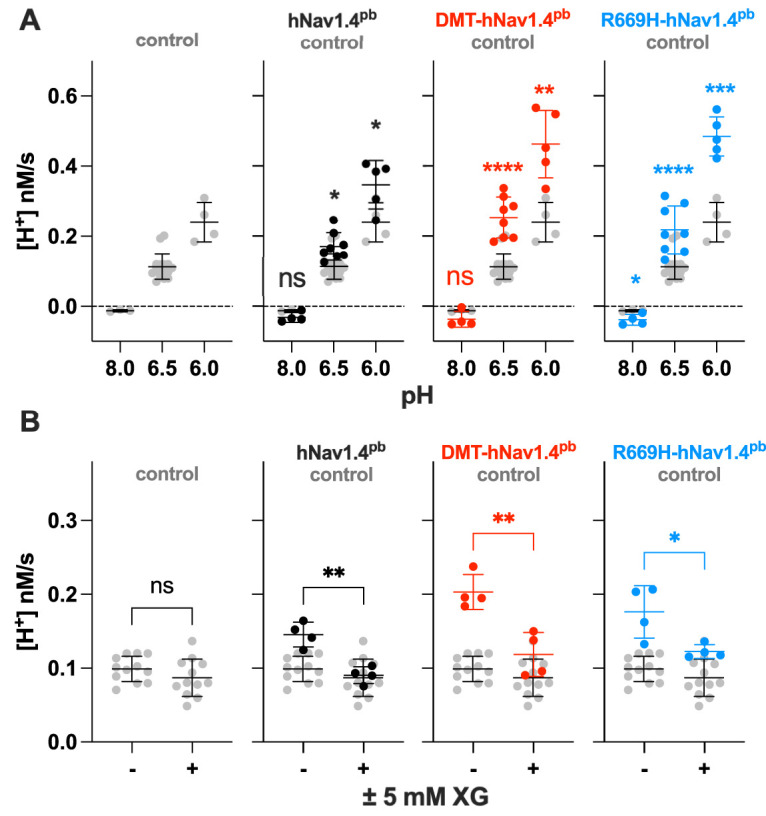
**Effects of pH gradients or a gating pore blocker on proton transport.** (A) Summary of H^+^ transport rates measured under different pH gradients in HEK293T-based cells co-expressing mKir2.1 and hNav1.4^pb^ (black) hNav1.4^pb^ (black), DMT-hNav1.4^pb^ (red) or R669H-hNav1.4^pb^ (blue) compared with those in control cells expressing mKir2.1 alone (gray). H^+^ transport was initiated by rapidly decreasing extracellular K^+^ concentrations (4–0.8 mM) and by changing pH values (from 7.4 to 8.0, 6.5 or 6.0). Dashed horizontal lines indicate a H^+^ transport rate of 0 nM/s. (B) Summary of H^+^ transport rates measured in the presence and absence of the gating pore blocker 1-(2,4-xylyl) guanidinium (XG) at 5 mM. Transport of H^+^ was initiated by rapidly decreasing extracellular K^+^ concentrations (4–0.8 mM) and pH values (from 7.4 to 6.5) as described for Fig. 4. For A and B, protein expression was induced by 1 µg/ml doxycycline. Horizontal bars indicate the mean±s.d. Significance was assessed by using Student's *t*-test. ns, not significant (*P*≥0.05). *P<0.05; **P<0.01; ***P<0.001; ****P<0.0001.

Before starting the assay, cells were suspended in a solution containing 4 mM K^+^ at pH 7.4. Proton transport assay was initiated by automated rapid injection of a low-pH buffer without K^+^ to each well (see ‘Proton transport assay’ in Materials and Methods) that reduced the external K^+^ concentration from 4 to 0.8 mM and the pH from 7.4 to 6.5. These changes enhanced the electric transmembrane potential and the inward H^+^ chemical gradient and, thus, synergistically augmented the gating pore currents in HypoPP model cells. Fig. 4C and Fig. S3 show results of proton transport assays performed for hNav1.4^pb^ and DMT-hNav1.4^pb^ in a doxycycline-dependent manner, i.e. using one well per one doxycycline concentration. The H^+^ transport rates [nM/s] were determined from data points collected within the first 20 min; of those, initial slopes at time zero were calculated as described previously (Wasano et al., 2020). The doxycycline-dependent H^+^ transport rates determined side-by-side for hNav1.4^pb^ and DMT-hNav1.4^pb^ are summarized in Fig. 4D (left panel). Cells expressing mKir2.1 alone served as negative control (0.15±0.03 nM/s, mean±s.d., *n*=9, indicated by horizontal line and gray underlay in Fig. 4D). We also quantified expressions of hNav1.4^pb^ and DMT-hNav1.4^pb^ by dividing mTq2 fluorescence (F_mTq2_) by the optical density of cells at 660 nm (OD_660_) (Fig. 4D, right panel). We found that the protein expression started to saturate at higher doxycycline dosages (>1 µg/ml) and that a high protein expression rendered cells unhealthy, as indicated by a decrease in cell doubling time). In light of these observations, we decided to determine and compare H^+^ transport rates among the hNav1.4^pb^ constructs by using data collected from cells treated with 1 µg/ml doxycycline (Fig. 4E). Consistent with the electrophysiological data shown in Figs 2 and 3, our optical assay successfully detected statistically significant increases in H^+^ transport rates for DMT-hNav1.4^pb^ and R669H-hNav1.4^pb^ (Fig. 4D,E). The hNav1.4^pb^-like small transport rate determined for R672H-hNav1.4^pb^ (Fig. 4E, left panel) may be at least partly ascribed to reduced total expression of the construct (Fig. 4E, right panel). To further demonstrate the ability of our optical method to measure H^+^ gating pore currents, we repeated the SNARF-based proton transport assay for DMT-hNav1.4^pb^ and R669H-hNav1.4^pb^ under different pH gradients. As summarized in Fig. 5A, augmentation of the inward H^+^ gradient (at between pH 6.5 and pH 6.0) increased the observed transport rates, whereas a reversed pH gradient (pH 8.0) resulted in reduced transport in the opposite direction (efflux of H^+^), confirming that our optical assay, indeed, monitors H^+^ flux. We also found that H^+^ transport rates are significantly reduced by the gating pore blocker 1-(2,4-xylyl) guanidinium (XG) (Sokolov et al., 2010), in cells expressing hNav1.4^pb^ constructs (Fig. 5B).

**Fig. 6.**
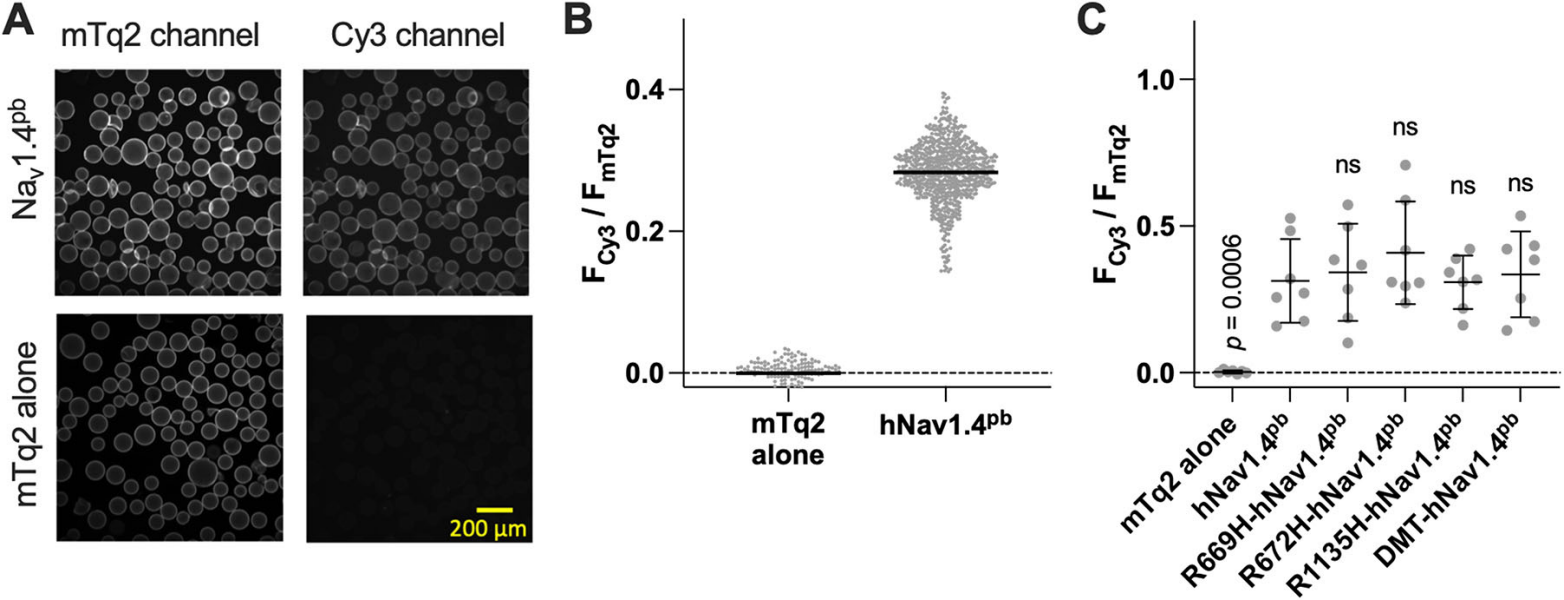
**Quantification of membrane targeting efficiencies of the hNav1.4^pb^ constructs.** (A) HEK293T-based cells stably expressing expressing mTq2-tagged hNav1.4^pb^ or mTq2 alone were treated with the membrane-impermeable amino-reactive fluorescent probe sulfo-Cyanine3 NHS ester (Cy3), washed and lysed. Detergent extracted mTq2 was captured using anti-mTq2 affinity beads. Images of the beads in mTq2 and Cy3 channels are shown. (B) Fluorescence intensities in the cyan (F_mTq2_) and red (F_Cy3_) channels were determined using images of the beads, and their ratios, which indicate the efficiency of cell membrane targeting, plotted. Plotted as examples are the results for mTq2-tagged hNav1.4^pb^ and mTq2 alone (negative control). Horizontal solid lines indicate the means. (C) Summary of cell membrane-targeting efficiencies. Horizontal solid lines indicate the means, error bars indicate ±s.d. One-way ANOVA with Dunnett's multiple comparison test. ns, not significant (*P*≥0.05).

In addition to quantifying the total protein expression (Fig. 4D,E, right panels), we also quantified the cell membrane targeting efficacies of the hNav1.4^pb^ constructs because the total protein expression (F_mTq2_/OD_660_) does not tell us how much Nav1.4 protein is actually targeted to the cell membrane, thus, contributing to H^+^ transport. An observed difference in H^+^ transport rate could be due to a change in unitary transport rate or cell membrane targeting efficacy. To distinguish these possibilities, we treated cells with an excessive amount of sulfo-Cyanine3 NHS ester (hereafter referred to as Cy3), lysed the cells with mild detergent-containing lysis buffer (see Materials and Methods), and pulled down using anti-GFP-conjugated beads (GFP-selector) that captures mTq2. mTq2 fluorescence (F_mTq2_) indicates total mTq2-tagged protein expression, whereas Cy3 fluorescence (F_Cy3_) indicates the amount of cell membrane-targeted population. Thus, the fluorescence ratio F_Cy3_/F_mTq2_ provides the cell membrane targeting efficacy. As expected, cells expressing hNav1.4^pb^ showed both Cy3 and mTq2 fluorescence (Fig. 6A, top panels), whereas those expressing mTq2 alone (negative control) did not (little or no Cy3 fluorescence, Fig. 6A, bottom panels), resulting in clearly distinguishable F_Cy3_/F_mTq2_ ratios (Fig. 6B). We performed this Cy3-based cell membrane targeting assay for the hNav1.4^pb^ constructs used in this study (*n*=7) and found that all of them target the cell membrane similarly (Fig. 6C). Therefore, the relatively larger hyperpolarization-induced inward currents (Fig. 3A) and H^+^ transport rates (Fig. 4) found in cells expressing DMT-hNav1.4^pb^ or R669H-hNav1.4^pb^ compared to those expressing hNav1.4^pb^ should be ascribed to increased unitary gating pore currents induced by these missense mutations but not to increased targeting of the cell membrane.

Our optical assay results are in line with those obtained in whole-cell patch clamp experiments, providing a proof-of-concept for the mammalian cell line-based optical screen platform described here. This study suggests that our optical assay system can be used to efficiently screen for gating pore blockers and, thus, find pharmacological solutions for HypoPP and other channelopathies caused by gating pore proton currents.

## DISCUSSION

Gating pore currents are small ionic-leak currents that do not pass through the canonical ion translocating pores of ion channels. *Xenopus* oocytes have been commonly used to electrophysiologically identify and characterize these small leak currents. However, mammalian proteins should – preferably – be expressed in mammalian cell lines, as they express mutated proteins of interest at physiological temperature. Also, mammalian cell lines have the great advantage to easily accommodate an increased culture size for high-throughput applications. In this study, we have generated stable HEK293T-based HypoPP model cell lines to co-express one of the four hNav1.4^pb^ mutants (i.e. R669H-hNav1.4^pb^, R672H-hNav1.4^pb^, R1135H-hNav1.4^pb^ or the DMT-hNav1.4^pb^), the Na^+^ channel β1 subunit and mKir2.1 by using the Sleeping Beauty transposon system. By using whole-cell patch clamp technique, we confirmed that gating pore currents induced by R669H-hNav1.4^pb^ and DMT-hNav1.4^pb^ are electrophysiologically detectable in HEK293T cells comprising hyperpolarized cell membrane potentials, i.e. below −70 mV, that these leak currents are mediated by proton ions and that the membrane potential – ranging from −65 mV to −95 mV – can be controlled by changing the extracellular K^+^ concentration when mKir2.1 is co-expressed. Based on these findings, we developed a plate reader-based assay that fluorometrically monitors H^+^-mediated gating pore currents. The observed sensitivity of optically determined transport activities to the transmembrane pH gradient and the gating-pore blocker XG, supports the ability of our plate reader-based assay to monitor gating pore proton currents mediated by certain HypoPP2-associated Nav1.4 variants.

The background proton transport activity detected in control cells expressing mKir2.1 alone may be ascribed to endogenous channels/transports and/or exchangers expressed in HEK293T cells, such as K^+^ channels or solute carrier family proteins (Rouillard et al., 2016). Heterologous expression of mKir2.1 might also contribute to background proton transport activity. In fact, to suppress background current, the K^+^-channel blocker tetraethylammonium (TEA) has been used in a previous study measuring gating pore currents (Moreau et al., 2015a). Since our optical assay relies on K^+^ conductance, TEA or any other K^+^ channel blocker could not be used. However, it was reassuring that this background proton transport activity was barely sensitive the gating-pore blocker XG (Fig. 5B) and, thus, was distinguishable from XG-sensitive proton currents. Nevertheless, it was unexpected that another control cell line, co-expressing mKir2.1 and hNav1.4^pb^, showed XG-sensitive proton transport activity. A previous study has examined the effect of the E761K mutation on cation conductance, albeit without examining the effect on proton conductance (Schlief et al., 1996). It is evident that the pore-blocking E761K mutation abolished the Na^+^ conductance of Nav1.4 (Figs 2A and 3A) but, possibly, not completely for H^+^ conductance. Our results showed that hNav1.4^pb^ can be slightly permeable to proton and that this small residual current is not negligible when the small gating pore currents are detected optically. However, proton transport rates of DMT-hNav1.4^pb^ and R669H-hNav1.4^pb^ were statistically larger compared to that of hNav1.4^pb^ control (Fig. 4), assuring the gating pore current measurements at least for these two HypoPP-hNav1.4^pb^ constructs.

In contrast to R669H-hNav1.4^pb^ and DMT-hNav1.4^pb^, neither R672H-hNav1.4^pb^ nor R1135H-hNav1.4^pb^ induced significant proton transport compared to hNav1.4^pb^ control (Fig. 4). These negative observations corroborate with the small leak currents observed at negative potentials for R672H-hNav1.4^pb^ and R1135H-hNav1.4^pb^ in whole-cell patch clamp experiments (Fig. 3A). Previous studies using COVC techniques in *Xenopus* oocytes report that R669H generates proton-selective transport most likely through the Grotthuss hopping mechanism (Struyk and Cannon, 2007), whereas gating pores generated by the R672H or R1135H mutations conferred relatively low proton specificity (Groome et al., 2014; Struyk et al., 2008). It is possible that the absence of significant gating pore currents in R1135H-hNav1.4^pb^ may be ascribed to both the structural–functional property of the arginine residue at position 1135 (Arg^1135^) and our assay system, which utilizes mKir2.1 and low K^+^ to hyperpolarize the cell membrane. The molecular mechanism to generate gating pores has not been fully elucidated but replacement of a voltage-sensing arginine residue located in S4 with a smaller amino acid might collapse the hydrophobic seal within the VSD. It is conceivable that the formation of a gating pore upon either hyper- or depolarization depends on the location of these voltage-sensing arginine residues within the VSD, i.e. R1, R2 and R3 within the S4 of hNav1.4 (Fig. S4). In fact, it has been shown that mutation of R1 (i.e. Arg^669^) or R2 (i.e. Arg^672^) in domain II of Nav1.4 exhibited gating pore currents upon hyperpolarization, whereas mutation of R3 (i.e. Arg^675^) in domain II of Nav1.4 exhibited gating pore currents upon depolarization (Sokolov et al., 2008; Gosselin-Badaroudine et al., 2012a). Interestingly, gating pore currents induced by mutation of Arg^1135^ (i.e. R3 of domain III) has shown inconsistent results: one report showed passing of protons and other cations at hyperpolarized as well as depolarized membrane potentials (Groome et al., 2014), while another study showed that this happened only at hyperpolarized membrane potentials (Zaharieva et al., 2016). It is also important to note that depolarized states of ion channels, i.e. relaxed or slow inactivated state, affect the voltage dependence of gating pore currents (Sokolov et al., 2010; Villalba-Galea et al., 2008). These factors need to be taken into account when measuring gating pore currents under depolarized conditions.

We propose to use the stable cell line expressing R669H-hNav1.4^pb^ and optical proton transport assay established in this study to rapidly screen for gating pore-blocking small molecules at relatively high concentrations and, subsequently, define their binding specificities and affinities by the COVC system – but only for promising candidates. If one seeks compounds that have inhibitory effects comparable to 5 mM XG with type I error rate of 0.05 and statistical power of 0.9, approximately eight replicates would be required for R669H-hNav1.4^pb^ (computed on the basis of the result shown in Fig. 5B with SD ∼0.03 nM/s). Considering the microplate reader-based format, these values are reasonably small. The latter, relatively time-consuming, electrophysiological efforts are crucial, especially because the optical assay would not distinguish inhibitory effects on mKir2.1 vs gating pore current mediated by hNav1.4 variants, and because binding kinetics of a promising compound identified at pH 6.5 might be different at a physiological pH, although there is uncertainty regarding pathophysiological pH in HypoPP. The large surface area of the cell membrane, which allows excessively high expression of R669H-hNav1.4^pb^ protein, and the easiness with which transmembrane electric potential and extra/intracellular solutions in the COVC system can be controlled, enable the electrophysiological determination of binding affinities and specificities with high precision. A *Scn4a^R669H^* (mNav1.4^R663H^) knock-in mouse model has been generated and confirmed to manifest the HypoPP2 phenotype (Wu et al., 2011). This HypoPP2 mouse model can be used for defining the clinical efficacies and examining potential adverse effects of promising gating pore blockers. In any case, our optical assay using R669H-hNav1.4^pb^ is of high clinical relevance, as R669H is one of the first mutations identified to be associated with HypoPP2 and one of the most frequently found in patients with HypoPP2 (Bulman et al., 1999; Sasaki et al., 2020).

In this study, we introduced only two ion channels – mKir2.1 and hNav1.4 – to generate an HEK293T-based HypoPP model cell line (Fig. 1C) because our focus was on gating pore currents anticipated to be induced by HypoPP-associated hNav1.4 variants. It should be noted that this simplified model does not recapitulate the overall HypoPP pathological conditions, such as the paradoxical depolarization observed in HypoPP muscle cells (Groome et al., 2014; Rüdel et al., 1984). Intricate physiological processes, such as the excitation–contraction coupling in skeletal muscle and the propagation of action potential in excitable cells, have been successfully replicated by using HEK293T cell lines (Kirkton and Bursac, 2011; Nguyen et al., 2016; Perni et al., 2017). Therefore, our HypoPP model can be further elaborated to recapitulate the broad HypoPP pathological conditions when additional channel/transporter components, such as Na^+^-K^+^-Cl^−^co-transporters, Na^+^-K^+^-ATPases or the CLC-1 (also known as CLCN1) chloride channel, are introduced into our HypoPP-model cells. Combined with *in silico* mathematical modeling, such *in vitro* model cells would provide significant pathophysiological insights underlying HypoPP.

Our optical proton transport assay might also be applicable for other disease-associated mutations found in VSDs of other voltage-gated ion channels, such as Cav1.1 that is relevant in HypoPP1; Nav1.5 – whose α-subunit is encoded by SCN5A – and which is relevant in dilated cardiomyopathy (Gosselin-Badaroudine et al., 2012b; Moreau et al., 2018); Cav2.1 – whose α-subunit is encoded by *CACNA1A* – and which is relevant in the familial hemiplegic migraine type 1 and/or episodic ataxia type 2 (Friend et al., 1999; Tantsis et al., 2016); KCNQ2, which is relevant in autism (Gamal El-Din et al., 2021); and others. Thus, future application of our non-electrophysiological recording system for gating pore currents should not be limited to HypoPP-associated channel variants.

## MATERIALS AND METHODS

### Molecular biology

cDNA coding for wild-type human SCN4A C-terminally conjugated to mTurquise2 (mTq2), followed by the self-cleaving peptide P2A sequence to mediate ribosomal skipping and SCN1B (SCN4A-mTq2-P2A-SCN1B) was subcloned into expression vector pSBtet-Pur (addgene #60507) (Kowarz et al., 2015) by using the NEBuilder HiFi DNA Assembly Cloning Kit (NEB). The missense mutation E761K that blocks the canonical ionic pore (Schlief et al., 1996), was introduced to SCN4A using KOD-Plus mutagenesis kit (TOYOBO, Osaka, Japan). The resulting construct SCN4A^E761K^ (hNav1.4^pb^) served as a control for gating pore current (Fig. 1A, gray). Hypokalemic periodic paralysis (HypoPP)-associated mutations R669H, R672H and R1135H were then introduced to hNav1.4^pb^ by using the KOD-Plus mutagenesis kit to generate R669H-hNav1.4^pb^, R672H-hNav1.4^pb^, R1135H-hNav1.4^pb^ and R669H/R672H (DMT)-hNav1.4^pb^. Wild-type mouse Kir2.1 (mKir2.1) was a generous gift from Dr Yoshihiro Kubo. The cDNA coding for mKir2.1 was subcloned into pSBbi-Bla (Addgene #60526) using the NEBuilder HiFi DNA Assembly Cloning Kit (NEB).

### Generation of stable cell lines

HEK293T-based stable cell lines that express hNav1.4^pb^ or its mutants in a doxycycline-inducible manner or that constitutively express mKir2.1 were generated using the aforementioned pSB vectors as previously described in detail (Kowarz et al., 2015), except that Lipofectamine 3000 (Thermo Fisher Scientific, Waltham, MA, USA) or ViaFect™ Transfection Reagent (Promega) was used for transfection. The cells were selected in a medium containing 1 µg/ml of puromycin or 10 µg/ml of blasticidin.

### Electrophysiology

Expression of hNav1.4^pb^ and its variants was induced by adding 3 µg/ml of doxycycline directly to the culture medium one day prior to recordings. The cells were then placed on 12 mm glass coverslips for the patch clamp recording. Gating pore currents obtained from HEK293T cells were recorded using whole-cell patch clamp technique. Recordings were made using Axopatch 200B (Molecular Devices. San Jose, CA, USA). Data acquisition and analysis were performed using Digidata 1550B (Molecular Devices, San Jose, CA, USA) and pCLAMP 11.1 software (Molecular Devices, San Jose, CA). Glass microelectrodes were heat-polished and used with resistances between 4.9 MΩ and 6.3 MΩ in N-methyl-D-glucamine (NMDG) solution or 1.6 MΩ and 2.1 MΩ in Ringer's solution. For gating pore current measurements in NMDG solution, the electrode solution contained 120 mM NMDG, 10 mM HEPES and 10 mM EGTA pH 7.4. Bath solution contained 120 mM NMDG, 2 mM CaCl_2_ and 20 mM MES buffer pH 6.5. For gating pore currents measurements in Ringer's solution, the electrode solution contained 105 mM KCl, 35 mM NaCl, 10 mM EGTA and 10 mM HEPES pH 7.4. The bath solution contained 140 mM NaCl, 4 mM KCl, 2 mM CaCl_2_, 1 mM MgCl_2_, 5 mM glucose and 20 mM MES (pH 6.5). Recordings were performed at room temperature, at 23-25°C. After achieving the whole-cell configuration, the membrane potential was held at 0 mV. gating pore current was measured by giving a 70 ms step pulse in 10 mV steps between −170 mV and +40 mV from a holding potential of 0 mV without any subtraction. Averaged values obtained between 65 ms and 68.5 ms were obtained for each step pulse voltage and plotted. Linear leak currents were subtracted manually by using currents obtained by step-pulses ranging from −20 mV to +20 mV (Fig. 2B). Raw gating pore current data are often corrected for gating currents, which are proportional to the amount of voltage-gated proteins expressed in the cell membrane. However, since we did not know how the missense changes introduced in this study would affect the gating charge movement of the hNa1.4 protein, and since it is technically challenging to precisely measure gating currents of Nav1.4 in cell lines, we did not correct gating pore current data in this study (Figs 2 and 3). Note that we quantified expression of the hNav1.4^pb^ constructs in the cell membrane by using mTq2 and Cy3 fluorescence (see examples in Figs 4 and 6), finding that protein expression was unlikely to affect our statistical conclusion in Fig. 2. We transfected cells with plasmids expressing hNav1.4^pb^ or its variants and performed experiments side by side. We repeated experiments (at least three sets) and confirmed reproducibility.

The membrane potential of HypoPP model cells was measured by using the Vm tracking mode. The electrode solution contained 105 mM KCl, 35 mM NaCl, 10 mM EGTA and 10 mM HEPES pH 7.4. The bath solution contained 140 mM NaCl, 0.8 mM or 4 mM KCl, 2 mM CaCl_2_, 1 mM MgCl_2_, 5 mM glucose and 20 mM MES pH 6.5. In the perfusion experiment shown in Fig. 3B, we confirmed expression of mKir2.1 by measuring its signature inward-rectifying Kir2.1 current by using the voltage-clamp mode. Ionic currents mediated by mKir2.1 were measured by giving a 70 ms step-pulse in 10 mV steps between −130 mV and +40 mV from a holding potential of 0 mV without any subtraction (Fig. 3B).

### Proton transport assay

HEK293T-based stable cell lines that co-express mKir2.1 and hNav1.4^pb^ constructs were established as described above. For proton transport assay, cells were cultured in 12-well plates. Expression of hNav1.4^pb^ constructs was induced by increasing concentrations (0–10 µg/ml) of doxycycline one day before the experiment, and cells were dissociated with Cell Dissociation Buffer (cat. no.: 13150016, Thermo Fisher Scientific). Cells were then loaded with the pH indicator SNARF-4F (cat. no.: S23921, Thermo Fisher Scientific) in 100 µl of wash buffer containing 140 mM NaCl, 4 mM KCl, 2 mM CaCl_2_, 1 mM MgCl_2_, 20 mM HEPES pH 7.4 for 30 min at room temperature. Thereafter, cells were washed once with and resuspended in 50 μl wash buffer, and transferred to 96-well plates (∼1.5×10^5^/well). Proton transfer assay was initiated by automated injection of 200 µl low-pH buffer without K^+^ (140 mM NaCl, 2 mM CaCl_2_, 1 mM MgCl_2_, 2.625 mM pH-unadjusted MES) in a Synergy Neo2 plate reader equipped with dual top photomultiplier tubes (Agilent/BioTeK). The fluorescence intensities, F_1_ and F_2_ (Fig. 4A), of SNARF-4F were measured in a time dependent manner (every 5 s for 1 h). The same assay was also performed in the presence of 5 mM 1-(2,4-xylyl) guanidinium (XG) mesylate that had been synthesized and described in a previous study (Jehasse et al., 2021). To examine the pH-dependence of the proton transport, 2.625 mM MES in the injected solution (200 µl) was replaced with either 5.29 mM Tris base or 5.54 mM MES to attain pH 8.0 or pH 6.0, respectively, after mix.

### Cell membrane targeting assay

Cells were seeded on 6-well plates and expression of mTq2-tagged hNav1.4^pb^ constructs was induced by addition of 3 µg/ml doxycycline for 1 day. Cells were washed once with PBS and, per well, 2 ml of the fluorescent dye sulfo-Cyanine3 NHS ester (10 µM) (Cy3; Lumiprobe) dissolved in ice-cold PBS were added and incubated for 30 min at 4°C. The reaction was stopped by addition of 200 µl of 100 mM glycine. Cells were then collected and lysed on ice in 500 µl of lysis buffer (150 mM NaCl, 20 mM HEPES pH 7.5, 1 mM EDTA, 20 mM DDM, 1 mM DTT and 50 µg/ml leupeptin). The lysate was centrifuged at 16,000 ***g*** for 5 min at 4°C and 5 µl of GFP selector slurry (NanoTag Biotechnologies) was added to the supernatant, followed by incubation for 30 min at 4°C with end-over-end mixing using a rotator. Bound proteins were collected alongside a GFP selector by brief centrifugation and observed under a fluorescence microscope (Leica DM IRB). Merged images of GFP selectors in cyan and red channels were analyzed using the image processing package FIJI (Schindelin et al., 2012) to determine the fluorescent signal intensities of mTq2 and Cy3.

### Statistical analyses

Statistical analyses were performed using Prism (GraphPad software), Origin (OriginLab) and JMP (JMP Statistical Discovery). Student's *t*-test was used for comparisons between two groups. One-way ANOVA combined with the Tukey's or Dunnett's post hoc test was used for multiple comparisons. In all statistical analyses, *P*<0.05 was considered significant.

## Supplementary Material

10.1242/dmm.049704_sup1 Supplementary information
