# Supplementary material for: Optical measurement of gating pore currents in hypokalemic periodic paralysis model cells
Source: Dis Model Mech. 2023 Jun 27;16(6):dmm049704. doi: 10.1242/dmm.049704 (PMC10320719; doi:10.1242/dmm.049704)
Supplement: Supplementary information [file dmm-16-049704-s1.pdf]

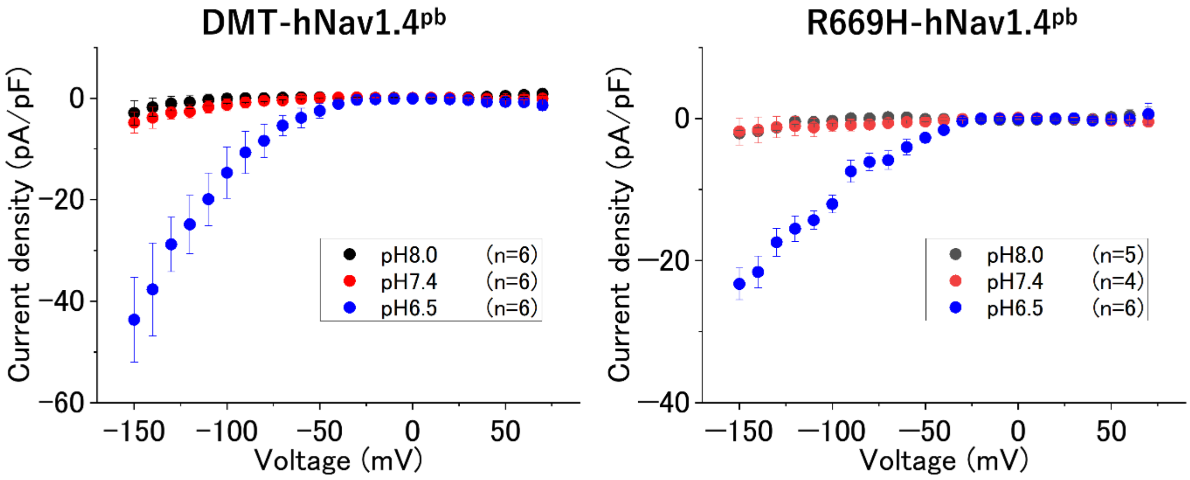

Fig. S1. Current density-voltage relationships of DMT-hNav1.4^{pb} (left) and R669H-hNav1.4^{pb} (right). The I-V data shown in Fig. 2D were corrected for the cell membrane capacitance. Error bars indicate SE.

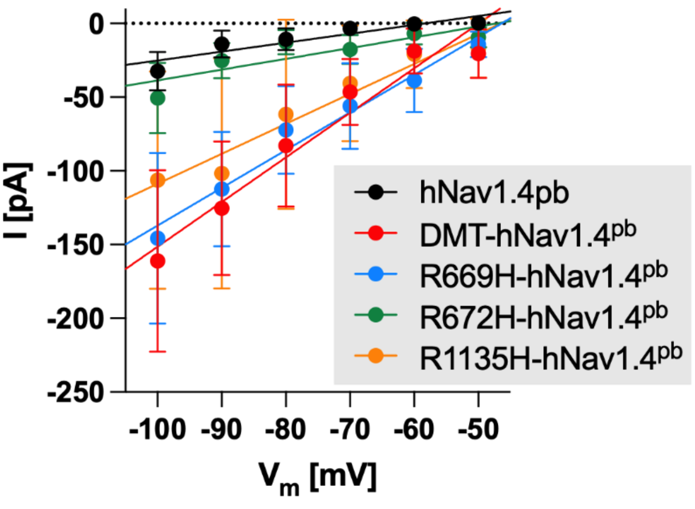

***F*-test** (data from -100 mV to -50 mV)

hNav1.4^{pb} vs. DMT-hNav1.4^{pb}: p = 0.0003
hNav1.4^{pb} vs. R669H-hNav1.4^{pb}: p = 0.0022
hNav1.4^{pb} vs. R672H-hNav1.4^{pb}: p = 0.7142 (ns)
hNav1.4^{pb} vs. R1135H-hNav1.4^{pb}: p = 0.1162 (ns)

Fig. S2. Linear regression analyses for Fig. 3A. Linear regressions were performed for the I-V data shown in Fig. 3A between -50 mV and -100 mV. Differences in the slopes (compared to that of hNav1.4^{pb} control) were assessed by *F*-tests. The *p* values were as indicated.

Disease Models & Mechanisms • Supplementary information

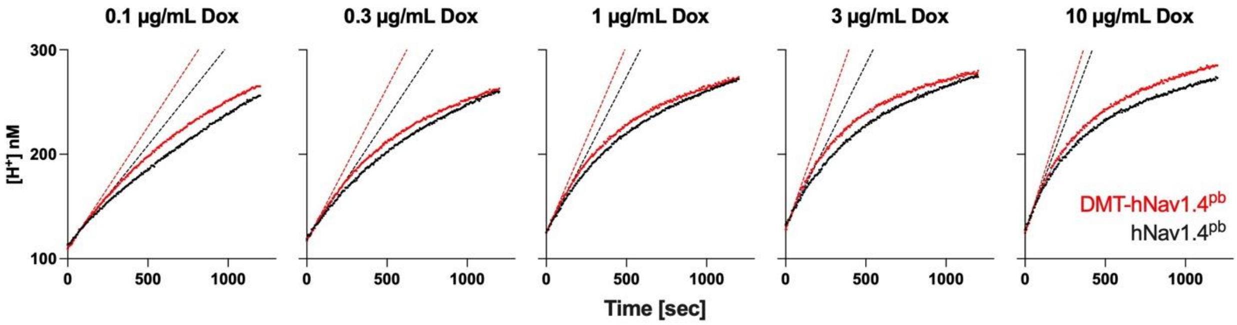

Fig. S3. Examples of H^+ transport assay for hNav1.4^{pb} and DMT-hNav1.4^{pb}. The data shown in Fig. 4C are replotted for comparing the initial transport rates between hNav1.4^{pb} vs. DMT-hNav1.4^{pb} at five different doxycycline concentration conditions. The broken lines indicate the initial rates.

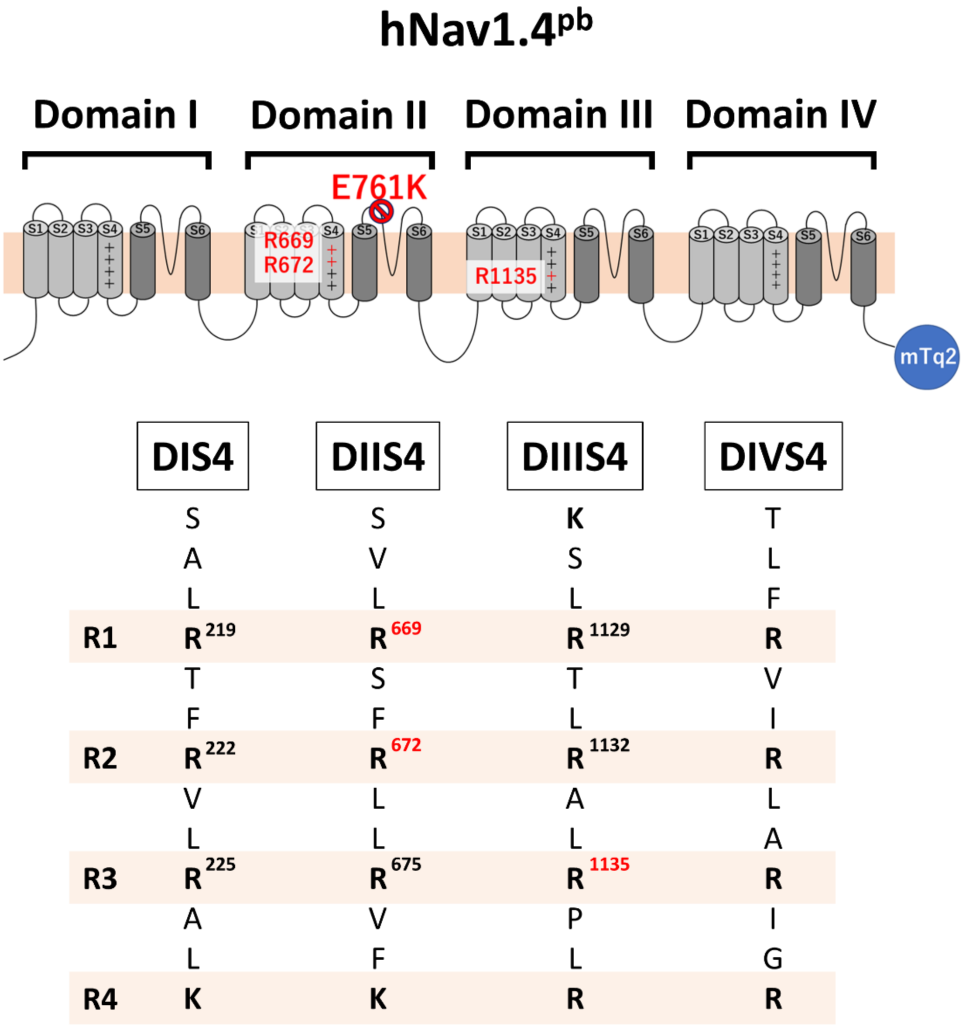

Fig. S4. Gating charges in segment 4 (S4) in hNav1.4.

Top: Schematic representation of human Nav1.4. D, domains (I-IV). Bottom: Amino acid sequence of S4 in each domain of human Nav1.4. Mutations used in this study are shown in red.

Table S1. A list of periodic paralysis-associated missense variants identified in VSD of Cav1.1

	Mutation	Position in VSD	GP experiments	Reference
HypoPP1	R528C	D2R1	No	(Yang et al., 2014)
HypoPP1	R528G	D2R1	Yes (Oocyte)	(Wu et al., 2018)
HypoPP1	R528H	D2R1	Yes (Oocyte)	(Wu et al., 2018)
			Yes (mouse model)	(Wu et al., 2012)
HypoPP1	V876E	D3	Yes (mouse myofiber with electroporation)	(Fuster, Perrot, Berthier, Jacquemond, Charnet, et al., 2017)
HypoPP1	R897S	D3R1	Yes (Oocyte)	(Wu et al., 2021)
HypoPP1	R897K	D3R1	Yes (Oocyte) †	(Kubota et al., 2020)
HypoPP1	R900G	D3R2	Yes (Oocyte)	(Wu et al., 2021)
HypoPP1	R900S	D3R2	Yes (Oocyte) †	(Wu et al., 2021)
HypoPP1	R1239G	D4R1	No	(Ptáček et al., 1994)
HypoPP1	R1239H	D4R1	Yes (mouse myofiber with electroporation)	(Fuster, Perrot, Berthier, Jacquemond, & Allard, 2017)
PPP*	R1242S	D4R2	No	(Wang et al., 2021)
NormoPP**	R1242G	D4R2	Yes (GLT cell line)	(Fan et al., 2013)

*Primary periodic paralysis. **Normokalemic periodic paralysis. † Gating pore current was not detected in these functional studies.

Disease Models & Mechanisms • Supplementary information

Table S2. A list of periodic paralysis-associated missense variants identified in VSD of Nav1.4

	Mutation	Position in VSD	GP experiments	Reference
HypoPP2	R219K	D1R1	Yes (Oocyte) †	(Kubota et al., 2020)
HypoPP2	R222W	D1R2	Yes (Oocyte)	(Männikkö et al., 2018)
HypoPP2	R669H	D2R1	Yes (Oocyte, mouse model)	(Mi et al., 2014; Sokolov et al., 2007; Struyk & Cannon, 2007; Wu et al., 2011)
HypoPP2	R672C	D2R2	Yes (Oocyte)	(Struyk et al., 2008)
HypoPP2	R672G	D2R2	Yes (Oocyte)	(Männikkö et al., 2018; Mi et al., 2014; Sokolov et al., 2007, 2010; Struyk et al., 2008)
HypoPP2	R672S	D2R2	Yes (Oocyte)	(Struyk et al., 2008)
HypoPP2	R672H	D2R2	Yes (Oocyte)	(Sokolov et al., 2007, 2010; Struyk et al., 2008)
NormoPP*	R675G	D2R3	Yes (Oocyte)	(Sokolov et al., 2008)
NormoPP*	R675Q	D2R3	Yes (Oocyte)	(Sokolov et al., 2008)
NormoPP*	R675W	D2R3	Yes (Oocyte)	(Sokolov et al., 2008)
HypoPP2/NormoPP*	R1129Q	D3R1	No	(Hong et al., 2010)
HypoPP2	R1132Q	D3R2	Yes (Oocyte)	(Francis et al., 2011; Männikkö et al., 2018)
HypoPP2	R1132G	D3R2	No	(Brugnoni et al., 2022)
HyperPP**	R1135C	D3R3	Yes (Oocyte)	(Groome et al., 2014)
HypoPP2	R1135H	D3R3	Yes (Oocyte)	(Groome et al., 2014)

*Normokalemic periodic paralysis. **Hyperkalemic periodic paralysis. † Gating pore current was not detected in this functional study.

Disease Models & Mechanisms • Supplementary information

References

Brugnoni, R., Canioni, E., Filosto, M., Pini, A., Tonin, P., Rossi, T., Canavese, C., Eoli, M., Siciliano, G., Lauria, G., Mantegazza, R., & Maggi, L. (2022). Mutations associated with hypokalemic periodic paralysis: from hotspot regions to complete analysis of CACNA1S and SCN4A genes. *Neurogenetics*, *23*(1), 19–25. https://doi.org/10.1007/S10048-021-00673-2

Fan, C., Lehmann-Horn, F., Weber, M. A., Bednarz, M., Groome, J. R., Jonsson, M. K. B., & Jurkat-Rott, K. (2013). Transient compartment-like syndrome and normokalaemic periodic paralysis due to a Cav1.1 mutation. *Brain*, *136*(12), 3775–3786. https://doi.org/10.1093/brain/awt300

Francis, D. G., Rybalchenko, V., Struyk, A., & Cannon, S. C. (2011). Leaky sodium channels from voltage sensor mutations in periodic paralysis, but not paramyotonia. *Neurology*, *76*(19), 1635–1641. https://doi.org/10.1212/WNL.0b013e318219fb57

Fuster, C., Perrot, J., Berthier, C., Jacquemond, V., & Allard, B. (2017). Elevated resting H $^{+}$ current in the R1239H type 1 hypokalaemic periodic paralysis mutated Ca $^{2+}$ channel. *The Journal of Physiology*, *595*(20), 6417–6428. https://doi.org/10.1113/JP274638

Fuster, C., Perrot, J., Berthier, C., Jacquemond, V., Charnet, P., & Allard, B. (2017). Na leak with gating pore properties in hypokalemic periodic paralysis V876E mutant muscle Ca channel. *The Journal of General Physiology*, *149*(12), 1139–1148. https://doi.org/10.1085/jgp.201711834

Groome, J. R., Lehmann-Horn, F., Fan, C., Wolf, M., Winston, V., Merlini, L., & Jurkat-Rott, K. (2014). NaV1.4 mutations cause hypokalaemic periodic paralysis by disrupting IIIS4 movement during recovery. *Brain*, *137*(4), 998–1008. https://doi.org/10.1093/brain/awu015

Hong, D., Luan, X., Chen, B., Zheng, R., Zhang, W., Wang, Z., & Yuan, Y. (2010). Both hypokalaemic and normokalaemic periodic paralysis in different members of a single family with novel R1129Q mutation in SCN4A gene. *Journal of Neurology, Neurosurgery, and Psychiatry*, *81*(6), 703–704. https://doi.org/10.1136/JNNP.2009.177451

Kubota, T., Wu, F., Vicart, S., Nakaza, M., Sternberg, D., Watanabe, D., Furuta, M., Kokunai, Y., Abe, T., Kokubun, N., Fontaine, B., Cannon, S. C., & Takahashi, M. P. (2020). Hypokalaemic periodic paralysis with a charge-retaining substitution in the voltage sensor. *Brain Communications*, *2*(2). https://doi.org/10.1093/braincomms/fcaa103

Männikkö, R., Shenkarev, Z. O., Thor, M. G., Berkut, A. A., Myshkin, M. Y., Paramonov, A. S., Kulbatskii, D. S., Kuzmin, D. A., Castañeda, M. S., King, L., Wilson, E. R., Lyukmanova, E. N., Kirpichnikov, M. P., Schorge, S., Bosmans, F., Hanna, M. G., Kullmann, D. M., & Vassilevski, A. A. (2018). Spider toxin inhibits gating pore currents underlying periodic paralysis. *Proceedings of the National Academy of Sciences of the United States of America*, *115*(17), 4495–4500. https://doi.org/10.1073/pnas.1720185115

Mi, W., Rybalchenko, V., & Cannon, S. C. (2014). Disrupted coupling of gating charge displacement to Na+ current activation for DIIS4 mutations in hypokalemic periodic paralysis. *The Journal of General Physiology*, *144*(2), 137–145. https://doi.org/10.1085/jgp.201411199

Ptáček, L. J., Tawil, R., Griggs, R. C., Engel, A. G., Layzer, R. B., Kwieciński, H., McManis, P. G.,

Santiago, L., Moore, M., Fouad, G., Bradley, P., & Leppert, M. F. (1994). Dihydropyridine receptor mutations cause hypokalemic periodic paralysis. *Cell*, *77*(6), 863–868. https://doi.org/10.1016/0092-8674(94)90135-X

Sokolov, S., Scheuer, T., & Catterall, W. A. (2007). Gating pore current in an inherited ion channelopathy. *Nature*, *446*(7131), 76–78. https://doi.org/10.1038/nature05598

Sokolov, S., Scheuer, T., & Catterall, W. A. (2008). Depolarization-activated gating pore current conducted by mutant sodium channels in potassium-sensitive normokalemic periodic paralysis. *Proceedings of the National Academy of Sciences of the United States of America*, *105*(50), 19980–19985. https://doi.org/10.1073/pnas.0810562105

Sokolov, S., Scheuer, T., & Catterall, W. A. (2010). Ion permeation and block of the gating pore in the voltage sensor of NaV1.4 channels with hypokalemic periodic paralysis mutations. *The Journal of General Physiology*, *136*(2), 225–236. https://doi.org/10.1085/jgp.201010414

Struyk, A. F., & Cannon, S. C. (2007). A Na+ channel mutation linked to hypokalemic periodic paralysis exposes a proton-selective gating pore. *The Journal of General Physiology*, *130*(1), 11–20. https://doi.org/10.1085/jgp.200709755

Struyk, A. F., Markin, V. S., Francis, D., & Cannon, S. C. (2008). Gating pore currents in DIIS4 mutations of NaV1.4 associated with periodic paralysis: saturation of ion flux and implications for disease pathogenesis. *The Journal of General Physiology*, *132*(4), 447–464. https://doi.org/10.1085/jgp.200809967

Wang, Q., Zhao, Z., Shen, H., Bing, Q., Li, N., & Hu, J. (2021). The clinical and genetic heterogeneity analysis of five families with primary periodic paralysis. *Channels (Austin, Tex.)*, *15*(1), 20–30. https://doi.org/10.1080/19336950.2020.1857980

Wu, F., Mi, W., Burns, D. K., Fu, Y., Gray, H. F., Struyk, A. F., & Cannon, S. C. (2011). A sodium channel knockin mutant (NaV1.4-R669H) mouse model of hypokalemic periodic paralysis. *Journal of Clinical Investigation*, *121*(10), 4082–4094. https://doi.org/10.1172/JCI57398

Wu, F., Mi, W., Hernández-Ochoa, E. O., Burns, D. K., Fu, Y., Gray, H. F., Struyk, A. F., Schneider, M. F., & Cannon, S. C. (2012). A calcium channel mutant mouse model of hypokalemic periodic paralysis. *Journal of Clinical Investigation*, *122*(12), 4580–4591. https://doi.org/10.1172/JCI66091

Wu, F., Quinonez, M., & Cannon, S. C. (2021). Gating pore currents occur in CaV1.1 domain III mutants associated with Hypopp. *Journal of General Physiology*, *153*(11). https://doi.org/10.1085/JGP.202112946/212609

Wu, F., Quinonez, M., DiFranco, M., & Cannon, S. C. (2018). Stac3 enhances expression of human CaV1.1 in Xenopus oocytes and reveals gating pore currents in HypoPP mutant channels. *The Journal of General Physiology*, *150*(3), 475–489. https://doi.org/10.1085/jgp.201711962

Yang, B., Yang, Y., Tu, W., Shen, Y., & Dong, Q. (2014). A rare case of unilateral adrenal hyperplasia accompanied by hypokalaemic periodic paralysis caused by a novel dominant mutation in CACNA1S: features and prognosis after adrenalectomy. *BMC Urology*, *14*(1). https://doi.org/10.1186/1471-2490-14-96

Disease Models & Mechanisms • Supplementary information
